# Incidence and associated factors of perioperative hypothermia in adult patients at a university-based, tertiary care hospital in Thailand

**DOI:** 10.1186/s12871-023-02084-2

**Published:** 2023-04-25

**Authors:** Mingkwan Wongyingsinn, Varut Pookprayoon

**Affiliations:** Department of Anesthesiology, Faculty of Medicine Siriraj Hospital, Mahidol University, Bangkok, 10700 Thailand

**Keywords:** Inadvertent perioperative hypothermia, Post-anesthesia care unit, Intraoperative temperature monitoring

## Abstract

**Background:**

Inadvertent perioperative hypothermia is an unintentional drop in core body temperature to less than 36 °C perioperatively and is associated with many negative outcomes such as infection, a prolonged stay in a recovery room, and decreased patient comfort.

**Objective:**

To determine the incidence of postoperative hypothermia and to identify the associated factors with postoperative hypothermia in patients undergoing head, neck, breast, general, urology, and vascular surgery. The incidences of pre- and intraoperative hypothermia were examined as the intermediate outcomes.

**Materials and methods:**

A retrospective chart review was conducted in adult patients undergoing surgery at a university hospital in a developing country for two months (October to November 2019). Temperatures below 36 °C were defined as hypothermia. Univariate and multivariate analyses were used to identify factors associated with postoperative hypothermia.

**Results:**

A total of 742 patients were analyzed, the incidence of postoperative hypothermia was 11.9% (95% CI 9.7%-14.3%), and preoperative hypothermia was 0.4% (95% CI 0.08%-1.2%). Of the 117 patients with intraoperative core temperature monitoring, the incidence of intraoperative hypothermia was 73.5% (95% CI 58.8–90.8%), and hypothermia occurred most commonly after anesthesia induction. Associated factors of postoperative hypothermia were ASA physical status III-IV (OR = 1.78, 95%CI 1.08–2.93, *p* = 0.023) and preoperative hypothermia (OR = 17.99, 95%CI = 1.57-206.89, *p* = 0.020). Patients with postoperative hypothermia had a significantly longer stay in the PACU (100 min vs. 90 min, *p* = 0.047) and a lower temperature when discharged from PACU (36.2 °C vs. 36.5 °C, *p* < 0.001) than those without hypothermia.

**Conclusion:**

This study confirms that perioperative hypothermia remains a common problem, especially in the intraoperative and postoperative periods. High ASA physical status and preoperative hypothermia were associated factors of postoperative hypothermia. In order to minimize the incidence of perioperative hypothermia and enhance patient outcomes, appropriate temperature management should be emphasized in patients at high risk.

**Registration:**

Clinical Trials.gov (NCT04307095) (13/03/2020).

## Background

Inadvertent perioperative hypothermia is an unintentional drop in core body temperature to < 36 °C (96.8 °F) in the perioperative setting, which is a common problem, particularly with general and regional anesthesia [1]. It has been implicated in negative outcomes in surgical patients, including prolonged effects of intraoperative anesthetic medication, increased insulin resistance, postoperative morbidity, delayed surgical wound healing, and prolonged stay in the recovery room and hospital [2, 3].

In the perioperative setting, heat loss can occur by radiation, conduction, convection, and evaporation; all of these mechanisms cause patients to have hypothermia [4]. Many guidelines provide recommendations for avoiding and managing perioperative hypothermia in surgical patients at each stage of the surgical journey, including the preoperative, intraoperative, and postoperative periods [2, 5–7].

Despite the fact that many active and passive warming systems are recommended in the perioperative setting to prevent hypothermia, such as using a preoperative warm blanket, optimizing ambient operative theater temperatures, and using various warming devices, the incidences of hypothermia remain high, up to 70%, particularly in the post-anesthesia care unit (PACU) [8–10]. Postoperative hypothermia alters physiology and drug metabolism, increases cardiovascular complications, shivering, nausea and vomiting, decreases patient comfort and satisfaction, prolongs recovery and PACU stay, and increases the cost of surgery [3, 11–13].

The overall incidence of postoperative hypothermia around the world ranged from 6–80% [1, 10, 12, 14–19]. Our hospital, a university-based, tertiary care hospital, reported a high incidence of postoperative hypothermia in PACU up to 45.4% in a study published in 2011 [19]. The outcomes also demonstrated age, preoperative body temperature, use of the warm blanket, abdominal irrigation, and type of surgery as the predictive factors of postoperative hypothermia [19]. Numerous studies have revealed that postoperative hypothermia is associated with older age, female, emergency surgery, higher American Society of Anesthesiology physical status, major surgical procedure or abdominal surgery, amount of intravenous or blood replaced, longer duration of anesthesia or surgery, operating room temperature, preoperative body temperature, and anesthetic technique [12, 20, 21]. Regarding the high incidence of postoperative hypothermia, various interventions and guidelines have been implemented at our hospital to prevent perioperative hypothermia in the last two decades. This study was initiated to examine the incidence of postoperative hypothermia in adult patients undergoing surgery at our university hospital after implementation of preventive measures. The incidences of perioperative hypothermia including in the preoperative and intraoperative periods were reported as the intermediate outcomes. The secondary objective is to identify the associated factors for postoperative hypothermia in the PACU.

## Materials and methods

### Study design and population

A retrospective review of the chart was performed after the registration of the clinical trial (NCT04307095) (13/03/2020) and the approval of the institutional review board (Si 040/2020) approval. In this study, adult patients who underwent head, neck, breast, general, urological, and vascular surgery with an anesthesia service were included. Patients who had a local anesthesia procedure with monitored anesthetic care, anesthetic time less than 30 min, an operation with intentional hypothermia or hyperthermia, admission to the PACU in less than 30 min, direct transfer to an intensive care unit or ward, or missing temperature data in the PACU were excluded.

### Hospital setting

Our hospital is a tertiary care hospital with 2154 beds that is affiliated with a university. The faculty comprises 25 departments that cover all medical areas. All 69 operating theaters provide surgical care for patients of all ages (general, pediatric, and geriatric patients) and all surgical specialties, including general surgery and subspecialties (pediatric surgery, neurological surgery, vascular surgery, plastic surgery, cardiothoracic surgery, urology surgery), orthopedic surgery, obstetrics and gynecology, otorhinolaryngology, and ophthalmology.

In the preoperative holding area, all patients are given a blanket and their core body temperature is measured using tympanic membrane thermometers. Patients whose temperatures are below 36 °C will be covered with an additional warm blanket or a forced-air warmer.

All patients receive passive insulation throughout the intraoperative period. Forced-air warmers, blood and intravenous fluid infusion warmers, and heated surgical tables are optional devices that can be used for intraoperative active warming. The anesthesiologists determine whether to measure a patient’s temperature and where to monitor. If the intraoperative temperature was being monitored, a record would be made every 15 min. Before patients are transferred from the operating theater, they are given two layers of a warmed blanket that are stored in a warming cabinet.

The body temperature of all patients is measured with tympanic membrane thermometers within 5 min of arrival in the PACU. If the temperature is less than 36 °C or the patient acknowledges feeling cold or is shivering, the forced-air warmer system is applied over the whole body. The temperature is measured repeatedly and the patients are queried about their thermal comfort every 5 min during warming. After the body temperature achieves 36 °C, it will be monitored every 15 min until the patients meet the discharge criteria (modified Aldrete score) and leave the PACU.

### Data collection

All patient data was retrospectively obtained from the hospital information technology program. One of the authors collected perioperative data from surgical notes, anesthetic records, and perioperative nursing records. Patient characteristics included age, sex, body mass index (BMI), and physical status as defined by the American Society of Anesthesiologists (ASA). Data on patients’ preoperative temperatures in the surgical preparation area were collected from preoperative nursing records.

Intraoperative details included the type of surgery (general, head, neck, and breast, urological and vascular surgery), intraabdominal surgery, anesthetic technique, duration of surgery, estimated blood loss, amount of fluid administered and blood component, amount of surgical irrigation fluid, and use of warming devices. The monitoring and intraoperative temperatures of the patients were recorded every 15 min. Data on postoperative temperatures were also collected in the PACU on arrival and discharge. In each period, patients with a temperature less than 36 °C were considered hypothermic [1]. Postoperative data included incidence of shivering and treatment; and length of stay in the PACU.

### Statistical analysis

All data were prepared and compiled using the Statistical Package for the Social Sciences program version 18.0 for Windows (SPSS Inc, Chicago, IL). Continuous variables were tested for normal distribution using the Kolmogorov-Smirnov and Shapiro-Wilk tests. Data were expressed as mean ± standard deviation (SD) or median [interquartile; IQR], and compared using the Student’s t-test or the Mann-Whitney U test, if appropriate. Categorical data were expressed as numbers (percentage) and compared using the Pearson Chi-square test or the Fisher exact test. Factors associated with postoperative hypothermia were analyzed using univariate analysis and the odds ratio with a 95% confidence interval (95%CI) for each variable was determined. Significant variables with a *p*-value of less than 0.2 from the univariate analysis were included in a multivariate binary logistic regression model and a *p*-value of < 0.05 was considered statistically significant.

The sample size was calculated based on the 7.27% incidence of postoperative hypothermia among our 275 PACU patients. With a 95% confidence interval of 7.27% ± 2%, a sample of 627 patients was required. Due to the retrospective cohort’s design, the sample size was increased to 815 in order to account for the estimated 30% of excluded patients and the possibility of missing or incomplete data. Regarding the secondary objective of assessing associated factors for hypothermia, there would be 59 patients with hypothermia out of 815 patients. Based on the rule of thumb of 10 EPVs (events per variable; event = hypothermia) in multiple logistic regression, 59 patients with hyperthermia would allow 6 associated factors in the multivariable analysis.

## Results

**Fig. 1:**
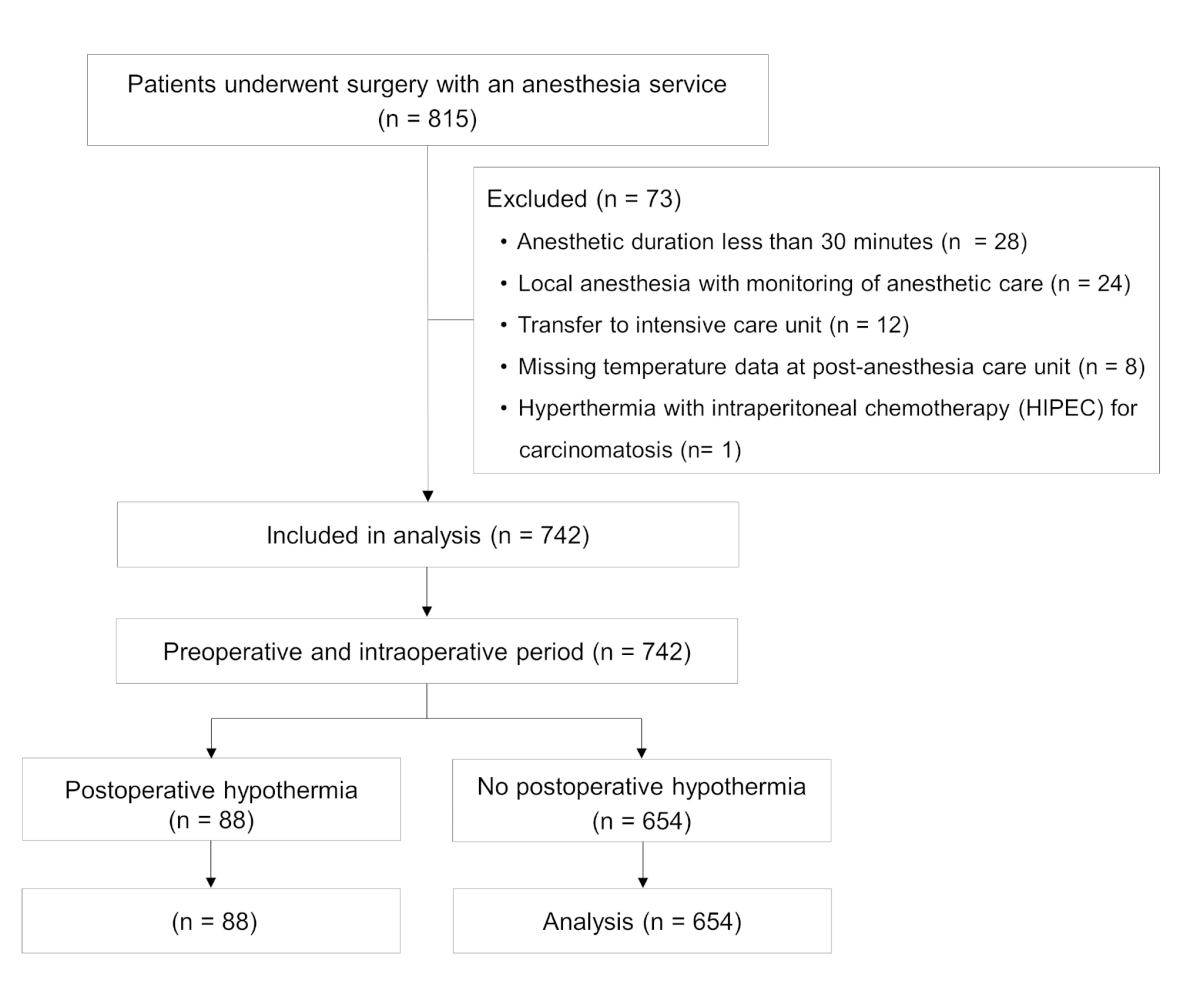
Study flow diagram

This study included 815 cases of electronic data recorded from patients who underwent surgery between October and November 2019. As a result of 73 cases being excluded from the study, a total of 742 cases were examined (Fig. 1).

### Incidence of postoperative hypothermia

**Fig. 2:**
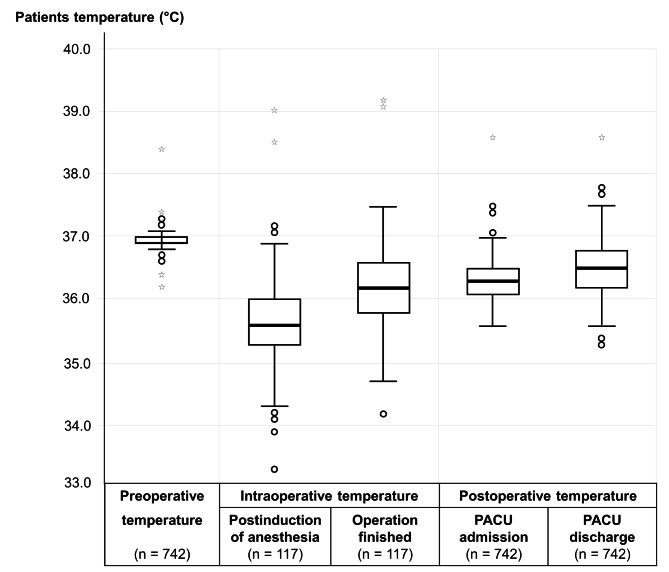
Preoperative, intraoperative, and postoperative temperatures

During the postoperative period in the PACU, 88 patients had hypothermia at admission and 6 patients still had core temperatures less than 36 °C when discharged. The incidence of postoperative hypothermia measured in the PACU was 11.9% with a 95% CI of 9.7–14.3%. The distribution of preoperative, intraoperative, and postoperative temperatures is presented in Fig. 2.

### Incidence of pre- and intraoperative hypothermia

**Fig. 3:**
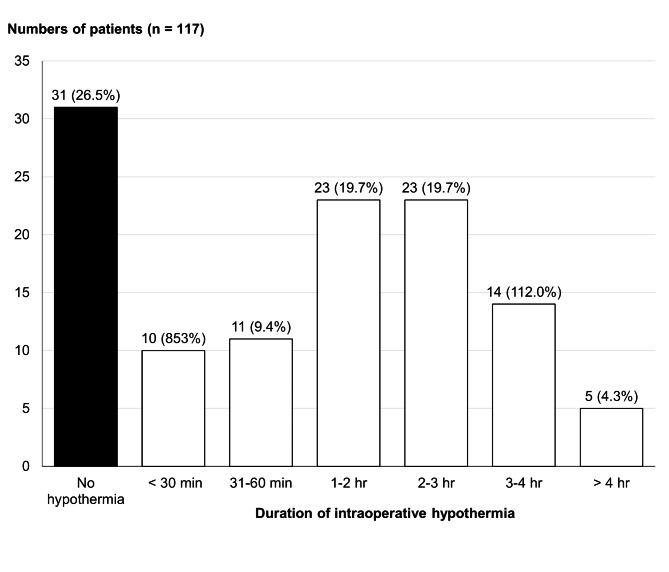
Duration of intraoperative hypothermia

The incidence of preoperative hypothermia measured in the prep area was 0.4% (3 patients) with a 95% CI of 0.08–1.2%. During the operation, 532 patients (71.7%) received forced-air warming, but only 121 patients (16.3%) had their temperature monitored intraoperatively, including core temperature monitoring (94 patients), skin surface monitoring (4 patients), and no record of temperature site monitoring (23 patients). After excluding 4 patients with skin surface monitoring, the overall incidence of intraoperative hypothermia in patients with temperature monitoring was 73.5% (95% CI 58.8–90.8%). 86 (73.5%) of the 117 patients reported hypothermia after induction of anesthesia. The duration of intraoperative hypothermia is presented in Fig. 3. 65 patients (55.6%) of these patients experienced hypothermia continuously for more than 60 min during an operation. However, the number of patients with hypothermia decreased to 33 (28.2%) when the operation was completed.

### Analysis of associated factors of postoperative hypothermia

The factors associated with postoperative hypothermia were investigated and shown in Table 1. Univariate analysis revealed that advanced age (≥ 60 years), ASA physical status III-IV, and preoperative hypothermia were strongly associated with postoperative hypothermia (*p* < 0.05), whereas factors potentially associated with postoperative hypothermia were type of surgery, intraoperative transfusion, intraoperative colloid use, and surgical irrigation fluid of ≥ 500 mL. A multivariate analysis adjusted for postoperative hypothermia risk factors revealed that ASA physical status III-IV was an independent predictor (OR = 1.78, 95%CI = 1.08–2.93; *p* = 0.023) and preoperative hypothermia was a significant risk factor (OR = 17.99, 95%CI = 1.57-206.89; *p* = 0.020) (Table 2).

**Table 1:** Factors associated with postoperative hypothermia

Item	Postoperative hypothermia		*p*-value
	Yes (n = 88)	No (n = 654)	
**Age (years)**			0.009
< 60	28 (8.4%)	305 (91.6%)	
≥ 60	60 (14.7%)	349 (85.3%)	
**Sex**			0.282
Male	46 (13.2%)	302 (86.8%)	
Female	42 (10.7%)	352 (89.3%)	
**Body mass index (kg/m** ^**2**^ **)**			0.388
< 18.5 (underweight)	8 (15.4%)	44 (84.6%)	
18.5–24.9 (normal weight)	39 (10.0%)	350 (90.0%)	
25-29.9 (overweight)	28 (13.0%)	187 (87.0%)	
≥ 30 (obese)	13 (15.1%)	73 (84.9%)	
**ASA physical status**			0.035
I	15 (10.8%)	124 (89.2%)	
II	35 (9.4%)	337 (90.6%)	
III	36 (16.9%)	189 (84.9%)	
IV	2 (33.3%)	4 (66.7%)	
**Type of surgery**			0.066
Head, neck, breast surgery	14 (7.9%)	164 (92.1%)	
Vascular surgery	11 (9.7%)	102 (90.3%)	
General surgery	30 (12.0%)	220 (88.0%)	
Urology	33 (16.4%)	168 (83.6%)	
**Intraabdominal surgery**	31 (13.7%)	195 (86.3%)	0.300
**Anesthesia technique**			0.624
General anesthesia	63 (12.1%)	456 (87.9%)	
Regional anesthesia	10 (9.1%)	100 (90.9%)	
Intravenous sedation	9 (15.8%)	48 (84.2%)	
Combined general–regional anesthesia	6 (10.7%)	50 (89.3%)	
**Preoperative hypothermia**	2 (66.7%)	1 (33.3%)	0.039
**Intraoperative hypothermia** (n = 121)	15 (17.4%)	71 (82.6%)	0.233
**Duration of surgery (min)**	67.5 [40-128.5]	75 [35-121.25]	0.636
**Estimated blood loss (mL)**	10 [5, 50]	15 [5, 52.5]	0.384
**Intraoperative transfusion**	4 (25%)	12 (75%)	0.110
**Intraoperative colloid administration**	3 (27.3%)	8 (72.7%)	0.132
**Intravenous fluid administration (mL)**	450 [250–700]	445 [250–750]	0.518
**Surgical fluid irrigation (mL)**	100 [0-1000]	65 [0-662.5]	0.189
**Forced air warming**	62 (11.7%)	470 (88.3%)	0.783
**Intraoperative temperature monitoring**	17 (14.0%)	104 (86.0%)	0.415

Data presented as number (percentage) or median [interquartile range].

**Table 2:** Univariate analysis and multivariate logistic regression of factors potentially associated with postoperative hypothermia

	Crude OR (95% CI)		*p*-value	Adjusted OR (95% CI)	*p*-value
**Age (Years)**					
< 60	1			1	
≥ 60	1.87 (1.17–3.01)	0.009		1.61 (0.98–2.67)	0.063
**ASA physical status**					
I-II	1			1	
III-IV	1.82 (1.15–2.86)	0.009		1.78 (1.08–2.93)	0.023
**Type of surgery**					
Head, neck, breast surgery	1			1	
Vascular surgery	1.26 (0.55–2.89)	0.168		0.77 (0.31–1.93)	0.579
General surgery	1.60 (0.82–3.11)	0.580		1.28 (0.37–2.57)	0.486
Urology	2.30 (1.19–4.46)	0.013		1.60 (0.80–3.20)	0.182
**Preoperative hypothermia**					
No	1			1	
Yes	15.19 (1.36-169.24)	0.039		17.99 (1.57-206.89)	0.020
**Intraoperative transfusion**					
No	1			1	
Yes	2.55 (0.80–8.08)	0.110		1.77 (0.50–6.25)	0.377
**Intraoperative colloid use**					
No	1			1	
Yes	2.85 (0.74–10.95)	0.132		2.13 (0.49–9.28)	0.312
**Surgical fluid irrigation (mL)**					
< 500	1			1	
≥ 500	1.55 (0.98–2.43)	0.059		1.45 (0.89–2.34)	0.133

Patients who had postoperative hypothermia when they arrived at the PACU stayed longer than those who did not (100 min [IQR 80–135 min] vs. 90 min [IQR 75–120 min], *p* = 0.047). In hypothermic patients, the average core temperature at discharge from the PACU was significantly lower than in normothermic patients (36.2 °C [IQR 36.1–36.5 °C] vs. 36.5 °C [IQR 36.3–36.8 °C], *p* < 0.001). However, the incidence of postoperative shivering in both groups was comparable (13.6% vs. 12.1%, *p* = 0.729), as was the number of patients receiving medications for shivering treatment (91.7% vs. 98.7%; *p* = 0.248).

## Discussion

The results of this study demonstrated that hypothermia still occurred in the postoperative period and occurred more frequently intraoperatively in adult patients undergoing surgery, particularly during the post-induction period. Despite efforts to actively and passively warm patients during the operation; 11.9% of patients arrived at the PACU with hypothermia. Preoperative hypothermia in a prep area was a risk factor for postoperative hypothermia. ASA physical status III-IV was another independent predictor of postoperative hypothermia.

Our study still reported high incidences of inadvertent hypothermia, especially in the intraoperative and postoperative periods. Although interventions such as warm blankets and forced air warming were applied to keep our adult patients comfortably warm before surgery at the normal temperature range of 36.0 to 37.5 °C [22]., a few patients still had preoperative hypothermia (0.4%) and 74.4% experienced intraoperative hypothermia. The number of patients who had intraoperative hypothermia was similar to a previous report (77%) in colorectal surgery within an enhanced recovery after surgery pathway from our hospital published in 2019 [23]. The overall incidences of intraoperative hypothermia around the world ranged from 30-50% [14, 16, 24]. but can reach up to 75% in some specific settings [15, 25]. Our results showed that the core temperature decreased rapidly during the first hour of anesthesia, which can be explained by several mechanisms, such as radiation and convection from the skin to the cold-temperature environment in the operating theater, conductive heat loss from body tissues to a large amount of unwarm intravenous fluid administration during anesthesia induction, and redistribution of heat from the core compartment to peripheral tissue due to vasodilating from anesthetic agents [5, 26, 27]. The active warming protocol and standardized maneuvers should be initiated early in the operative theater and before induction of anesthesia to prevent intraoperative hypothermia.

Intraoperative temperature monitoring is essential for clinicians to recognize and manage inadvertent hypothermia early. However, our study showed that patients who underwent intraoperative temperature monitoring only 16.3%, which was related to several studies with a temperature monitoring rate of 13.5–19.4% [28–30]. This study revealed that nearly 75% of patients have intraoperative hypothermia, which is a significant number. The high incidence may be due to anesthesiologists’ bias in patient selection, as they might only monitor the temperature in patients who were at risk for developing hypothermia. This bias can lead to an inaccuracy in the incidence of intraoperative hypothermia. Our incidence of intraoperative hypothermia decreased from 75.3% at the beginning of surgery to 28.2% when the operation was over. These results confirmed that intraoperative temperature monitoring is important to allow the detection of hypothermia and support immediate temperature management to promote normothermia [25, 31]. Additionally, core temperature monitoring at the nasopharynx, esophagus, and bladder is recommended for intraoperative use as it provides greater precision than skin temperature (forehead, axilla, and great toe) [32–34].

The incidence of postoperative hypothermia on admission to PACU was 11.9%. The incidence is lower than several studies (21-53.5%) [15, 17, 18]. and also less than a previous report from our hospital published in 2013 (45.4%) [19]. This result has been demonstrated as a significant improvement from various intraoperative patient temperature interventions performed following guidelines for the prevention and management of perioperative hypothermia, such as covering patients throughout the operation and exposing only the surgical field, warming patients intraoperatively using a forced air warming device, warming intravenous fluids when a volume of more than 500 ml was planned to administer, and warming all irrigation fluid [22, 27]. However, there is still an opportunity for improvements such as using humidified gases for anesthesia, temperature controlled mattress, surgical bed warming system, setting the appropriate ambient operating theater temperature at least 21 °C, or even encouraging intraoperative temperature monitoring to raise awareness of intraoperative hypothermia between surgeons, anesthesiologists, and nurses in the operators [22, 27].

The significant predictors of postoperative hypothermia in this study were ASA physical status III-IV and preoperative hypothermia. These results were consistent with various studies and practice guidelines [15, 19, 22, 27]. The probable explanation for ASA physical status is that coexisting medical diseases might delay the onset and diminish the efficacy of thermoregulatory vasoconstriction. Although multivariate logistic regression showed that advanced age (≥ 60 years) was associated with postoperative hypothermia (OR 1.61, 95% CI 0.98–2.67), there was no statistical significance (p = 0.063) as in previous studies [13, 18, 19]. Furthermore, ours did not demonstrate a significant association between postoperative hypothermia and body mass index [17]., type of surgery [13, 19, 22, 27]., anesthetic technique [22, 27]., duration of surgery and anesthesia [18]., intraoperative temperature monitoring [17]., volume of surgical irrigation fluid [19]., and use of warming devices [15, 17, 19]., as in the literature.

Our results showed a strong association between postoperative hypothermia and a longer stay in the PACU. The most common use of active warming systems in PACU is forced-air warming (e.g. Bair Hugger) which can increase an average core body temperature of 1.7 °C per hour [35]. This can explain why hypothermia patients may need more time to rewarm and achieve a target core temperature of 36.0 °C or higher. Although active skin surface warming was used during the postoperative period, the mean core temperature in hypothermic patients, when discharged from the PACU, was still significantly lower than those who were normothermia upon admission to the PACU (36.2 °C vs. 36.5 °C).

## Limitations

There are some limitations to this study. First, this study investigated adult patients who underwent general, head, neck, breast, vascular, and urological surgery only, it can be difficult to extrapolate our results to pediatric patients or patients undergoing other operations such as cardiothoracic, neurological, spine, or orthopedic surgery. Second, some intraoperative temperatures in our study were documented as a measure of skin surface, and some have no record of the temperature site. Although we imply that no record of the temperature site was a core temperature, this can lead to imprecise results of temperature records with a high standard deviation with respect to different methods of temperature assessment [33, 34]. Third, regarding the retrospective study, we have no record of operating theater temperature, which can be another associated factor of inadvertent hypothermia. The effect of ambient temperature on patients’ core temperatures has still been debated in many studies [16, 36]. A recent study concluded that the ambient intraoperative temperature has a small impact on the core temperature in patients who were warmed with forced air [36]. However, ambient temperature should be increased if necessary to maintain normothermia in high-risk patients. Forth, our sample size calculation, which was primarily based on the primary outcome, would cover only the estimated six independent variables. However, when ten events per variable (EPVs) are used, the 88 postoperative hypothermia events reported in this study can cover about nine independent variables with binary logistic regression analyses. We also identified an additional limitation, including that data on the long-term effects of postoperative hypothermia were not collected after discharge from the PACU.

## Recommendations

Our results support that perioperative medical professionals must be aware of perioperative hypothermia and create hospital policies to develop a guideline for the prevention and management of inadvertent perioperative hypothermia. The guideline should cover the identification of high-risk patients, accurate record of preoperative, intraoperative, and postoperative temperature with appropriate frequency and method, passive or active warming for indicated patients, and close monitoring.

Future studies could investigate other factors that were not included in this study that may contribute to perioperative hypothermia, including ambient environment temperature or the use of specific warming devices. More studies are needed to determine the best methods to measure temperature in all phases of perioperative patient care. Finally, future studies should investigate postoperative recovery, morbidity, and mortality of patients with perioperative hypothermia after discharge from the PACU.

## Conclusions

Inadvertent hypothermia still occurs in the perioperative setting, particularly in the intraoperative and postoperative periods. Patients with a high ASA physical status or who had preoperative hypothermia in a prep area were associated with postoperative hypothermia in the PACU. Meticulous temperature management should be emphasized in patients with high risk to prevent perioperative hypothermia and improve patients’ outcomes.

## Data Availability

The datasets generated during and/or analyzed during the current study are available from the corresponding author upon reasonable request.
